# Preverbal infants’ understanding of social norms

**DOI:** 10.1038/s41598-024-53110-3

**Published:** 2024-02-05

**Authors:** Moritz Köster, Robert Hepach

**Affiliations:** 1 Institute of Psychology, University of Regensburg, Sedanstraße 1, 93055 Regensburg, Germany; 2 Department of Experimental Psychology, University of Oxford, Oxford, UK

**Keywords:** Cultural evolution, Psychology and behaviour, Human behaviour, Cooperation

## Abstract

Social norms are foundational to human cooperation and co-existence in social groups. A crucial marker of social norms is that a behavior is not only shared, but that the conformity to the behavior of others is a basis for social evaluation (i.e., reinforcement and sanctioning), taking the *is*, how individuals usually behave, to an *ought*, how individuals should behave to be socially approved by others. In this preregistered study, we show that 11-month-old infants grasp this fundamental aspect about social norms already in their first year. They showed a pupillary surprise response for unexpected social responses, namely the disapproval and exclusion of an individual who showed the same behavior like others or the approval and inclusion of an individual who behaved differently. That preverbal infants link the conformity with others’ behavior to social evaluations, before they respond to norm violations themselves, indicates that the foundations of social norm understanding lie in early infancy.

## Introduction

Humans are unique in their capacity to establish and enforce social norms^1,2^, enabling human cooperation and co-existence in social groups^3,4^. Social norms are distinct from mere behavioral regularities by their social consequences, namely how individuals *ought* to behave to be socially approved and accepted by others. That is, social norms are established and maintained through social evaluation mechanisms^5^, the social reinforcement of behavior that conforms to the behavior of others and the social sanctioning of behavior that is non-conforming. Given their pervasive role for human everyday social interactions identifying and acquiring the norms of their social environment is a key developmental task from very early in life, allowing developing children to become competent and accepted members of their social group^6^.

Prevailing accounts posit that young children develop an understanding of social norms in their third year^7,8^. Around this age, children begin to enforce social norms by means of verbal protest or by correcting others’ behavior, when an individual does not behave in accordance with a previously established activity^9,10^. For example, 3-year-olds inferred a norm from a single demonstration of a novel and arbitrary action^10^. When one individual performed an action with a novel object, they protested when another individual performed a different action with the same object, even if the original action was not explicitly introduced as a rule (i.e., not requiring normative language). A first study showed inclinations to correct others’ behaviors in children as young as 18 months, when an interaction partner changed their behavior in a joint action context^11^. The authors interpreted this finding with regard to the joint action and propose that a more abstract normative stance, regarding the behavior of third parties, develops only after the second birthday, possibly developing from those commitments made in joint activities. To date, there is no evidence whether or not infants show an understanding of social norms in third parties even earlier, before they explicitly enforce social norms themselves.

Prior work demonstrated that young infants possess crucial socio-cognitive capacities^12–16^ and such capacities develop prior to behavioral manifestations^17^. For example, this has been shown in the case of early helping behavior, one of the earliest manifestations of infants’ triadic interaction capacities^18^. Here, it has been found that young infants in the first year of life expect others in need to be helped, as measured through their anticipatory looking behavior and looking time. Such expectations developed before infants themselves begin to help others in need. Furthermore, it has been shown that infants’ developing motor capacities and social interaction skills around 16 months moderated the link between their understanding of others’ needs (i.e., socio-cognitive capacity) and their own helping behavior towards an individual in need (i.e., behavioral manifestation). Similarly, it may well be that infants’ normative understanding develops considerably earlier than their verbal capacities and behavioral manifestations, namely their verbal and active protest when another individual is not adhering to a social norm (For a similar argument, see^19^).

In line with these considerations, critical conceptions about social group membership and joint activities have been documented in the first year already. For example, 8- to 12-month-olds expected the members of a group to act alike, by moving along similar movement trajectories^16^. Furthermore, already 6-month-olds associated the imitation of others’ behavior (making the same sound or movement) with their affiliative tendencies. They expected that the characters would join a specific group after imitating the movement or sound of this group^20^ (for complementary evidence in 16-month-olds, see^21^) and preferentially looked at imitators (vs. non-imitators), i.e., at those characters who conformed to the behavior of other members of a group^22^. Furthermore, young infants possess an understanding of social evaluations by others and socially evaluate others already in their first year in the case of prosocial and antisocial behaviors^13^. Recently it has even been shown that young infants punish undesirable social behaviors themselves, in the case of unfair resource distributions^23^ or harmful behavior towards others^24^.

However, a critical test for an early understanding of social norms would be whether infants, prior to their own normative protest, link the conformity or non-conformity of an individual with a novel and arbitrary behavior of others to the social evaluation by others, taking the *is*, how individuals usually behave, to an *ought*, that behaving like others is the basis for social evaluation^2,7^. That is, based on the studies reviewed above^13,16, 20–24^, do infants in their first year already understand that the conformity with the behavior of others has social consequences, conforming behavior being socially approved (i.e., reinforced), but non-conforming behavior being socially disapproved (i.e., sanctioned), which is a key marker of social norms^4,5^? Here it is crucial to test infants’ responses to novel, arbitrary, actions, to go beyond the social evaluations towards behaviors that have themselves positive connotations because they are supportive of others’ needs (e.g., helping versus hindering^13,25^). These behaviors may be associated with positive (versus negative) responses either naturally, or based on infants former learning experience. Infants may indeed understand the association between the conformity with others and their social responses, because of the pivotal importance for young infants to understand and adopt to the behavior of close others. Furthermore, social approval and disapproval are an integral part of infants’ experiences from early on. They themselves experience social evaluations in their interactions with close others, but also in interactions they observe between other individuals. Thus, the question arises, whether young infants already infer what ought to be done (i.e., what is right or wrong, as being associated with positive or negative social consequences), based on novel behaviors they observe in others.

**Figure 1:**
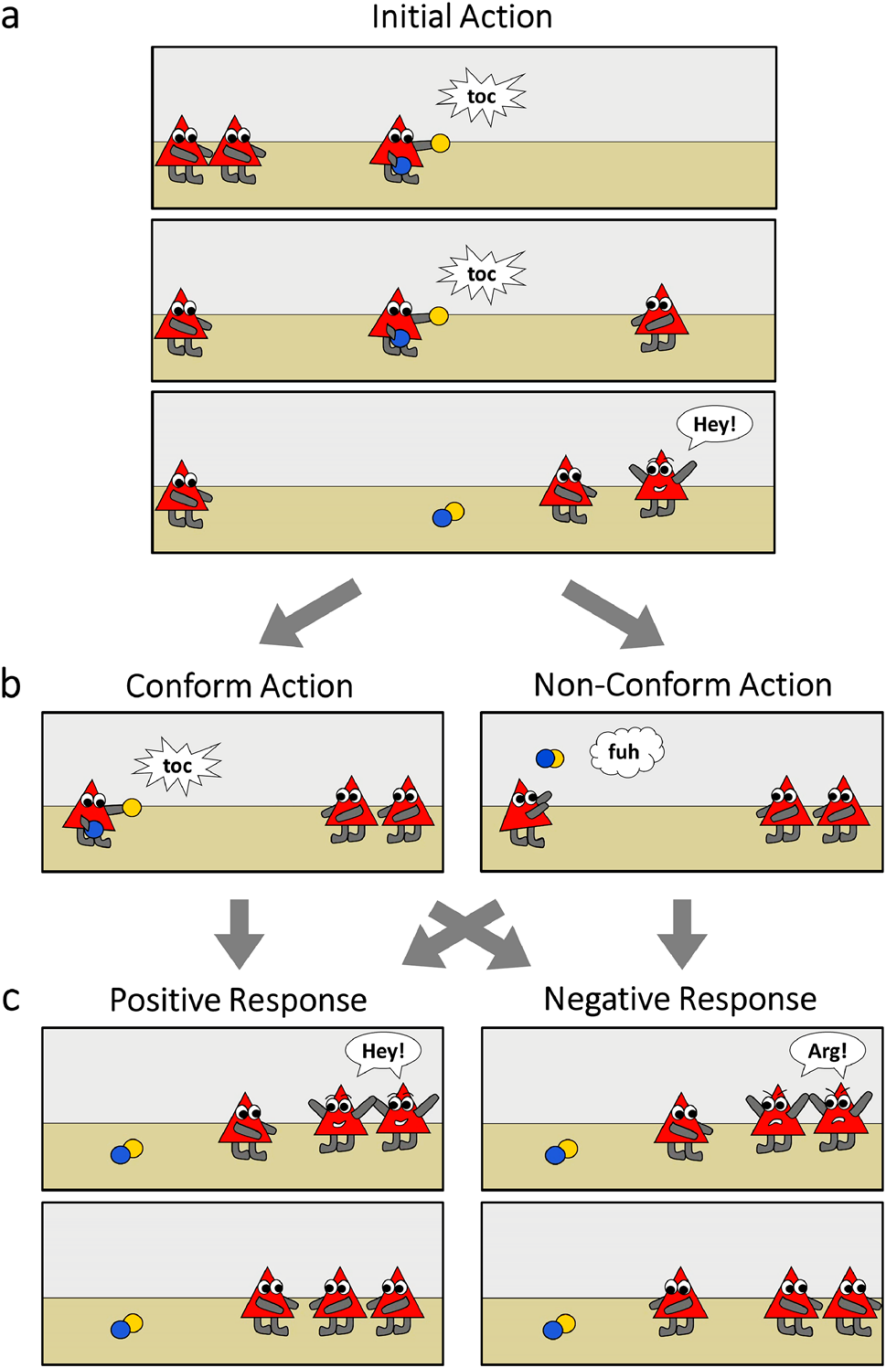
Animated picture stories presented to the infants. (**A**) In the build-up phase, a character performs an action with two objects (e.g., knocking 2 balls together, producing a “toc” sound). Then, a second character performs the same action and joins the first character, who responds positively (happy expression, “hey” sound). (**B**) The third character (the individual) shows either conform or non-conform behavior, by performing the same or a different action (e.g., throwing the balls up in the air, producing a “fuh” sound). (**C**) The character is then approved and included (happy expression, “hey” sound, moving towards the individual) or disapproved and ostracized (angry expression, “arg” sound, turning away from the individual). The final scene remained for 5 s. Note that picture stories were animated as movies and different characters and actions were presented (see Movies S1 to S4).

To address this question, here we conducted a preregistered study to test such an early understanding of social norms in their first year. Specifically, we presented 11-month-old infants with two characters performing an identical action, one after another, and that the first character responded positively to the action of the second character (build-up phase, Fig. 1A). Thereafter, a third character performed either the same (conforming [C]) or a different (non-conforming [NC]) action than the other two characters (Fig. 1B). In the critical scene, the third character approached the other two characters, who responded unidirectionally either with social approval (happy expression and inclusion [+]) or disapproval (angry expression and exclusion [−]; Fig. 1C). Infants viewed six different animated picture stories in each of the four experimental conditions (C+, C−, NC+, NC−; see Fig. 1 and see Movies S1 to S4) in 8 variants (see Fig. S1) and we recorded their pupil diameter and gaze behavior with an eye-tracker. Our decision to study 11-month-old infants was based on prior work suggesting that infants from this age onward understand more complex social interactions^20–22^.

Our hypothesis was that infants associate conforming and non-conforming actions of the third character with a positive or negative social evaluation by the other two characters, respectively. To test this hypothesis, we measured changes in their pupil diameter (i.e., pupil dilation), following the social response towards the third character. Increases in pupil diameter are a neurophysiological marker of a surprise response^26,27^ and, more generally, of higher cognitive demands associated with the experience of novel events^28^. Formerly, it has been shown that infants show greater pupil dilation in response to impossible physical events^29^ or incongruent social interactions^30–32^. Likewise, we measured infants’ looking times after the social reaction of the group (during a presentation of the final scene for 5 s; cf.^15^) as a violation of expectation measure. We predicted that already preverbal infants link the conformity with others’ behaviors to social evaluations and, consequently, that unexpected social reactions (C−, NC+) elicit a greater pupil dilation compared to the expected social reactions (C+, NC−), and likewise expected longer looking times for the unexpected versus expected social reactions.

Critically, the present experimental design allowed us to test whether infants were responding to perceptual novelty (new emotion or new action) or conceptual novelty (incongruent or congruent emotion-action coupling)^29^. That is, if infants responded to perceptual novelty, one would expect a main effect of emotion (i.e., higher pupil dilation and longer looking time in response to the negative emotion) and a main effect of action (i.e., higher pupil dilation and longer looking time in response to the non-conforming action). In this case, NC- would show the highest pupillary response. In contrast, if infants responded to the conceptual novelty, we  would expect an interaction effect of emotion and action (i.e., higher pupil dilation and longer looking time in response to incongruent emotion-action couplings). In this case, C- and NC+ should show the highest pupillary response.

Furthermore, we included a fixed number of non-social control trials, as a separate block, at the beginning of the session. This was preregistered design decision based on former studies^15,17^. In these trials two objects moved consistently for two times and then consistent or inconsistent at the third instance (i.e., changing the movement trajectory), and two characters responded positively or negatively to this non-social event (see Fig. S2). This was to test infants’ basic associations between consistent versus inconsistent events with a positive versus negative social response, to obtain initial evidence for whether or not infants’ differences in pupil diameter or looking times rests on more basic associations (about consistent events rather than conform actions).

## Results

**Figure 2:**
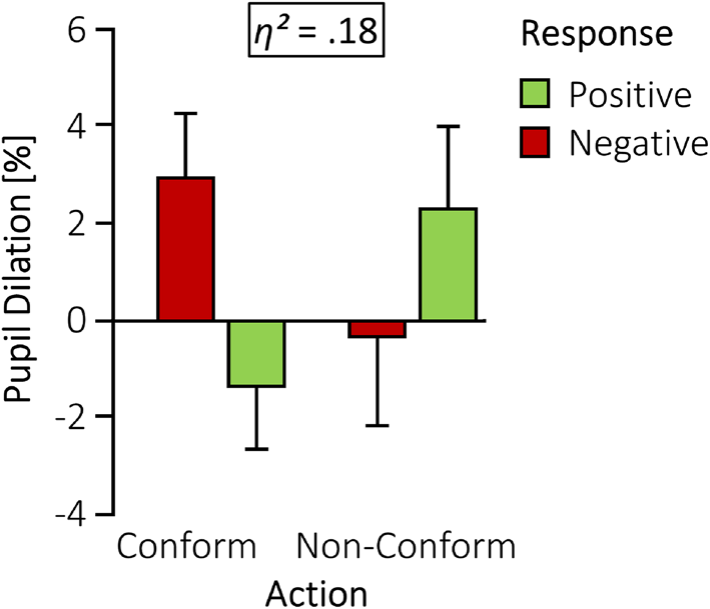
Infants’ pupil dilation in the experimental condition. Whiskers indicate standard errors. Infants’ pupil diameter increased for inconsistent social responses (C− and NC+), but not for consistent responses (C+ and NC−). This was indicated by the Action (conform, non-conform) × Response (positive, negative) interaction. The effect size is displayed two-sided *p* = .019.

Confirming our hypothesis, infants associated conforming actions (C+) of the third individual with social approval and inclusion by the other individuals, but non-conforming actions (NC−) with social disapproval and exclusion. This was indexed by infants’ pupillary surprise response when the other two individuals responded inconsistently, in contrast to the consistent responses (Action [C, NC] × Response [+, −] interaction: *F* [1, 28] = 6.17, *p* = 0.019): Their pupil diameter increased when they disapproved of the conforming individual (C−), and also when they approved the non-conforming individual (NC+; see Fig. 2). There were no main effects of Action or Response (both *p*s > 0.570). The magnitude of the interaction points to a medium to large effect (*pη*^*2*^ = 0.18).

**Figure 3:**
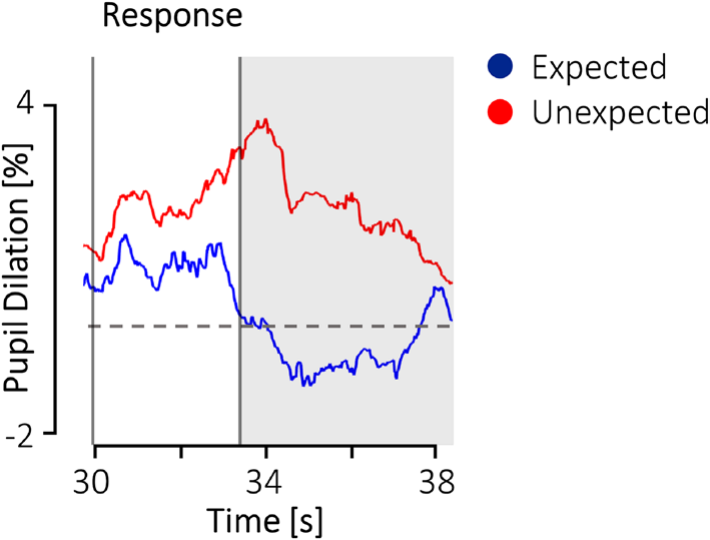
The temporal course of the pupillary response in the experimental condition. The response window (white area) indicates the period of the expected and unexpected reaction of the two individuals, towards the action of the third individual. The gray area indicates the time window included in the analyses.

The robustness of the Action × Response interaction effect was supported by a split-half reliability, which we conducted to substantiate that the pattern of results also holds for subsamples of all participants. Both subsamples revealed a similar effect size (sample A: *pη*^*2*^ = 0.20, sample B: *pη*^*2*^ = 0.17, see Methods). Furthermore, we explored the temporal course of the pupillary surprise response (see Fig. 3). This revealed that the difference in pupil diameter between expected (C+, NC−) and unexpected (C−, NC+) outcomes emerged during the response of the two individuals and peaked just after they turned away from (or towards) the third individual.

We did not find any significant interaction in infants looking times within the 5 s following the outcome screen (Action [C, NC] × Response [+, −] interaction: *F* [1, 29] = 1.34, *p* = 0.257, *pη*^*2*^ = 0.04). However, infants showed marginally longer looking times towards the positive social responses (main effect Response: *F* [1, 29] = 3.21, *p* = 0.083, *pη*^*2*^ = 0.10, see Table S1).

To test whether infants’ pupillary response data was related to the amount of time they spent looking at the screen during the crucial 5 s time window (i.e., whether the duration of time that infants did not look at the screen had an influence on the pupil dilation measure), we correlated the looking time recorded for each trial with the change in infants’ pupil diameter. This analysis was a luminance control analyses to rule out that changes in pupil size were related to the mere time infants looked at the illuminated screen (for similar control analyses, see^33^). This revealed that the overall amount of time infants fixated at the screen had was not directly related the assessed pupillary response measure, *r* = − 0.05, *p* = 0.391.

Further analyses of infants’ gaze behavior supported the idea that the action of the third individual was associated with the actions performed by the other two individuals. At the time the third individual performed an action, infants showed more referential gaze behavior towards the other two individuals, when they performed the same action (*M* = 47.0% of all trials included in the looking time analysis, *SE* = 5.7%), in contrast to the different, non-conform action (*M* = 31.9%, *SE* = 3.9%), paired t test: *t* [29] = 2.57, *p* = 0.016.

Critically, infants’ understanding of social norms may rely on more basic associations of the consistency of events with positive or negative responses. For an initial assessment of this possibility, we presented infants with a series of non-social trials, at the beginning of each session^15,16^. In these trials, two individuals were confronted with objects moving by themselves and which moved either consistently or inconsistently before the two characters responded positively or negatively (movements of the objects were matched to the movements of the identical objects in the experimental conditions; see Fig. S2). This analysis indicated that infants’ understanding about social norms was not based on this low-level mechanism. We found no Action (C, NC) × Response (+, −) interaction in infants’ pupil dilation (*F* [1, 19] = 0.38, *p* = 0.547) or looking times (*F* [1, 18] = 0.39, *p* = 0.543) and no main effect in any condition in both ANOVAs (all *p*s > 0.338; see Table S1 for the mean values). This was corroborated by considerably lower interaction effects in these trials (*pη*^*2*^ = 0.02 for both measures). An inspection of the temporal course of the pupil dilation in these trials indicated an increase in infants’ pupillary response during the initial reaction of the two individuals, but the potentially expected and unexpected condition did not differ for the responses of the two individuals (Fig. S4).

## Discussion

We found that 11-month-old infants showed a basic understanding of social norms, in the sense that the conformity to the behavior of others is the basis for social evaluation, social approval (i.e., reinforcement) of conforming behavior or social disapproval (i.e., sanctioning) of non-conforming behavior. These findings reveal an association that acting like others is the basis for social evaluation and inclusion, at a preverbal age, before infants can make use of normative language. This indicates that the ontogenetic foundations of human normativity may be present much earlier than previously assumed^7,8, 34, 35^ and complements previous findings that infants’ implicit and descriptive socio-cognitive capacities often precede their explicit manifestation, including their intentional^12^, moral^13,14^, and prosocial understanding^15,31^. However, this was only indicated by infants’ pupillary surprise response towards unexpected versus expected action-response combinations, but not in their looking time, as discussed below.

Importantly, the design of the present study closely resembles the standard procedure used to assess an understanding of social norms at a verbal age: After the presentation of a novel, arbitrary action by one individual, another individual performs a different action on the same object^10^. In the original paradigm, it is then tested whether children respond to non-conforming actions by verbal protest or active correction themselves. This reaction is usually found from around the third year and a widely accepted marker for infants’ understanding of social norms^7^. Here, we tested whether preverbal infants would already associate a non-conforming action to a negative social response, before they are able to verbally express a normative reaction themselves. We did so by testing their pupillary surprise response for social evaluations by others, which were consistent or inconsistent with the enforcement of a previously established behavior (i.e., a response to conceptually incongruent action-emotion couplings).

Critically one could argue that this association indicates a descriptive understanding of social norms, linking the conformity with the actions of others to social evaluations, rather than a prescriptive understanding of social norms, applying to other characters in other contexts. A prescriptive understanding of social norms may only be indexed by normative language, when verbal capacities have developed later in life, or when shown across different social contexts. However, we favor the perspective of an implicit normative understanding that emerges prior to verbal and active forms of protest.

The link between the pupillary response and the neurophysiological arousal system is well understood^26,27^ and pupil dilation has become an established measure for the surprise response in young infants^29–34^, likely indexing the increased cognitive control demands associated with novel events in the environment^28^. For the interpretation of the present findings, it is critical to note that the pupil diameter did not serve as a direct measure of infants’ own social evaluation or expectation. Instead, the pupillary surprise response served as a marker for a violation of infants’ expectations of others’ social evaluations in responses to an agent’s actions conforming or non-conforming with the actions of the group. We did not find main effects for the emotion expressed by the group or for the agent’s action which would have suggested a response to mere perceptual novelty. Instead, our findings suggest that infants responded to the conceptual novelty of the observed situations. Infants’ pupil diameter increased for inconsistent (incongruent) social reactions (C− or NC+), in contrast to consistent (congruent) reactions (C+ or NC−). To further probe the nature of infants’ expectations, future research could employ a different kind of paradigm, for example, one in which children’s anticipatory looking or own behavioral responses are recorded (see^24,36, 37^ for similar paradigms). This would help to disentangle whether the surprise response documented in the current study is a result of children expecting (i.e., anticipating) a specific response of the group, specifically whether the degree of pupil dilation change in response to the incongruent action-emotion couplings is associated with the degree of anticipation prior to viewing the group’s response.

Our main finding points to a medium to large effect size (*pη*^*2*^ = 0.18) and is based on a pre-registered sample size, design, and hypotheses. In addition, we did not find any effect in the non-social trials, indicating that infants did not show a surprise response for a positive reaction following inconsistent over consistent events, when these did not reflect a conform versus non-conform action performed by a third character. Although the non-social analysis was based on only one trial for each condition and a smaller sample (given that we included a single trial for each condition in the beginning, which was not watched by all infants^15,17^), the interaction effect in the pupillary response was very low (*pη*^*2*^ = 0.02), suggesting that the consistency of the events do not explain the present results. Notably, such a reading of our findings would not undermine that those infants have an understanding of social norms per se, instead, such an effect would have suggested that an early concept of social norms may rely on yet more principal mechanisms (i.e., a core understanding that the consistency of events is associated with a positive of negative social response).

We did not find an interaction effect in infants looking times. This may have different reasons. First, it has been argued that looking times are much more sensitive to perceptual novelty, rather than conceptual novelty, and that the pupil dilation measure is therefore more suited to detect conceptual violations^29^. Second, the lack of sensitivity of the looking time measure may also be due to the limited time window following the social response (5 s) in the repeated design. Although such short time windows were successfully applied in previous studies^15^, it is not a standard in the field and the looking time differences in this design have been replicated by one^17^, but not all replication attempts^38^. Finally, it has been found that infants’ looking times can be increased for consistent^39^ but also inconsistent social interactions^15^. Thus, the non-significant looking time results must be taken with caution, and we cannot rule out looking time differences in case the design was optimized for the assessment of looking times.

Another reading of our main finding may be that infants interpret the social responses displayed by the characters as affiliation or liking (and the opposite thereof^20–22^). Here, to mimic social evaluations, we maximized cues to index social disapproval versus approval (unidirectional exclusion versus inclusion, angry versus happy facial expression, and disapproving versus approving vocalizations; see Movies S1 to S4). However, along their own experiences of social evaluations, which start very early in life, we know from prior studies that infants in the age range tested here already grasp and show social evaluations themselves (e.g.,^13,23, 24^).

It should also be noted that simple associative learning of the build-up phase can hardly explain the present results, mainly because infants’ pupil dilation was decreased in NC− trials, where they saw a completely different action (NC) and social response (−) then in the build-up phase, separating perceptual from conceptual novelty^29^ (see introduction). In the present study, the perceptual features of all characters within one sequence were held identical, to avoid that condition differences could be elicited by any perceptual differences. In addition, prior studies have explicitly tested and shown that infants are sensitive to group membership^20^ and this manipulation would also be interesting with regard to infants’ normative understanding. However, the classical paradigm with children at a verbal age does likewise not include a group manipulation^10^, and we wanted to keep the design of the present study as simple and as parallel to the behavioral paradigm as possible.

Based on studies investigating young children’s normative protest, current theoretical accounts propose that an understanding of social norms only develops around age three^7,8^. However, the normative protest observed in older children may depend on additional developmental attainments, including their verbal capacities, the conception of oneself as a guardian of a norm, and the motivation to intervene, when another individual violates a norm. These capacities presumably emerge only after the first year, in the interaction of infants with their social environment^40,41^. Critically, a former study^11^ documented protesting behavior in 18-month-old toddlers in a joint action setting. While the authors argued this to be an early form of protest in the context of joint task engagement, rather than an understanding about social norms in third parties, the present study suggests that infants possess such an understanding already in their first year of life. For future research, it would be intriguing, to test whether young children’s earliest forms of protest rest on their (implicit) understanding of social norms. Towards this end, the present findings mark a crucial first step towards a better understanding of the developmental system that underpins the early ontogeny of human normativity (for a similar conception on early prosocial development, see^18^). That is, how young children’s capacities develop from an understanding of social norms to enforcing normative behavior themselves.

To conclude, we found that already preverbal infants show a much more profound understanding of social norms than previously assumed. An early sensitivity for social norms is fundamental to human cultural and moral development and may equip preverbal infants with a socio-cognitive compass (i.e., to predict the social consequences of their own and others behavior^42^). This may enable them to internalize the norms which exist in their social environment and, thereby, to become competent members of their social group from very early in life.

## Methods

### Subjects

Thirty 11-month-old infants (*M*_*age*_ = 11.49 months, range: 10.26–12.30 months) were included in the final analyses. All subjects were healthy and born full term. Fifteen additional infants were excluded due to insufficient eye-tracking data (see eye-tracking analysis). Infants came from urban middle-class families in Leipzig and were recruited from the local database. The study protocol was approved by the ethics committee of the University of Leipzig (Ref. 169/17-ek) and informed written consent was obtained from the mother of each infant prior to the participation in the study. All experiments were performed in accordance with relevant guidelines and regulations. We aimed for a final sample of *N* = 30 infants (see preregistration). This corresponds with former studies measuring phasic changes in pupil dilation in infants around a similar age, which used similar (or somewhat lower) samples sizes^43,44^. The study design, sample size, and hypothesis were pre-registered on aspredicted.com (https://aspredicted.org/sx6yd.pdf). The study was conducted between 03/2018 and 08/2018, and the manuscript was firstly submitted for publication in 03/2019.

### Experimental design

In an initial build-up phase, two characters performed an identical action with two objects, one after another (Fig. 1A; corresponding [start and end] times in the movies: 3.0–6.0 s, and 11.1–14.1 s; see Movies S1 to S4). The first character responded positively to the conform action of the second character (happy facial expression, saying “hey, hey, hey!”; 16.6–19.6 s) and the second character joined the first character by moving next to it. In the test phase, a third character either performed the same action like the other two characters (conform, C) or another action, different from the action of the two characters (non-conform, NC), while being observed by the other two characters (24.4–27.4 s; Fig. 1B). Subsequently, the third character approached the other two characters, who reacted either positively (+) or negatively (−; Fig. 1C), namely with a happy facial and vocal expression (“hey, hey, hey!” in a high pitch) and getting together with the third character or with an angry facial and vocal expression (saying “arg, arg, arg!”, in a low pitch) and ostracizing the third character by turning away (29.9–33.4 s).

Note that the social response combines several elements of social approval versus social disapproval, namely inclusion versus exclusion, happy versus angry facial expressions, as well as an approving versus disapproving sound. All elements were relevant to render the responses prototypical of social approval and disapproval (following^16^). Infants discriminate positive and negative facial expressions towards the end of their second year of life^45^. Likewise, previous studies demonstrated that actions of moving towards each other^13^ as well as facial expressions^46^ are also differentiated by infants (and elicit meaningful effects on measures of pupil dilation^25^) for geometrically shaped characters. In addition, while former studies used explicit verbal expressions of praise and admonishment^47^ here we created novel sounds to support the characters approval or disapproval. The expression of approval or disapproval was directed (unidirectionally) from the group towards the individual approaching the group. This was to maximize the concept of social evaluation and this design is distinct from the mere gathering^20,22^ or mutual affiliation processes displayed and tested in previous studies^21^. In essence, the present design added the crucial aspect of social approval or disapproval whereas previous work included mutual liking or involved the agent approaching the group. Critically, in a 2-by-2 design both responses were presented as expected as well as unexpected outcomes, such that perceptual differences between outcomes were controlled in the within-subjects design. The final scene was presented as a still-frame for 5 s at the end of each trial to assess infants’ pupil dilation and looking time in response to the different outcomes (33.4–38.4 s, see eye-tracking procedure). Overall, this resulted in a 2 Action (C, NC) × 2 Response (+, −) within-subjects design.

Six animated picture stories were presented in each of the four action and outcome combinations. Picture stories varied in the objects and actions performed by the characters as well as the characters’ shapes and colors (see Fig. S1 and Movies S1 to S4). Furthermore, characters walked in either from the left of the scene to the right or from right to left. These variations served to sustain infants’ attention and to avoid transfer effects across trials. Within each picture story, all characters were identical in shape and color, to ensure that only the performed actions may be the reason for a positive or negative response to the third character (i.e., no perceptual difference between the characters could serve as a cue for why a character was included or excluded). This resulted in 24 trials, all 6 picture stories with each of the four potential outcomes, all including the build-up and test phase. Note that we chose 24 trials, to maximize the variation in the sequences shown to the infants and to obtain a good number of valid trials per infant. Overall, the procedure lasted up to 20 min, which is towards the upper end of the attention span of infants around the first year (cf.^15^). However, the presentation was stopped when infants lost interest.

We presented the picture stories in a pseudorandomized order, counterbalancing the presentation of the different picture stories, the different outcomes (C+, C−, NC+, NC−) as well as the action that was performed by the first two characters and the third character, between subjects. In consequence, the identical action carried out by the third character could either be congruent or incongruent to the action of the first two characters. This counterbalanced procedure ensured that luminance and contrast values were perfectly controlled between conditions, first, because both actions were presented as expected or unexpected and, second, because both outcomes (+, −) could be expected (C+, NC−) and unexpected (C−, NC+).

In four non-social control trials, infants saw animated picture stories in which the two objects in the center of the scene moved by themselves (see Fig. S3). This was to test the basic idea whether infants associate consistent events with a positive and inconsistent events with a negative response. Specifically, the objects either moved consistently, showing the same movement, three times in a row, or moved inconsistently, showing the same initial movement for two times (1.4–4.4 s and 5.1–8.1 s), but a different, inconsistent movement thereafter, as a third movement (9.0–12.0 s). This was followed by a positive or negative reaction of the two characters (14.0–17.1 s). The non-social stimuli were very similar to the experimental trials, but the two characters were already at their final position and there was no third character. We opted for displaying only the objects and no additional shape besides the moving objects (e.g., a red triangle), because infants’ may even perceive simple shapes as intentional agents^48,49,39^. Critically, our goal with the control trials was not to perceptually parallel the test trials, but to optimize the stimuli to test the specific association between the consistency of physical events and social responses. To prevent associations between the shapes and the characters in the test trials, the control trials (four trials, one in each condition) were shown at the beginning of each session, prior to the test trials^15,16^.

### Eye-tracking procedure and analyses

Infants sat on their parent’s lap, while stimuli were presented on a 24-inch computer screen (at 1280 × 720 pixels of the full HD resolution), at a distance of 70 to 90 cm. Lights in the laboratory were dimmed. The luminance of the monitor was reduced to a minimum to control for strong effects of the luminance on the pupil diameter. The gaze of the subjects was tracked with a remote eye-tracker (Tobii X3-120; Tobii Technology, Stockholm, Sweden), at a sampling rate of 120 Hz. A 5-point calibration was used. Individual fixations were exported from Tobii Prolab (Tobii I-VT filter) and analyzed in MATLAB (R2017b).

#### Pupil dilation analyses

We calculated the percentage of baseline-corrected change in pupil diameter in response to the final scene, i.e., once the group’s action was completed (see Fig. 1). Specifically, and as outlined in our preregistration, we averaged the pupil diameter values recorded within the final scene of each trial (33.4–38.4 s) and divided this value by the mean baseline value of each respective trial. The baseline was defined as the time period just before the first experimental manipulation (see Fig. 1A, 21.1–23.1 s, see also^38^, for a similar choice of a 2 s baseline period). We subsequently averaged the percentage of change in pupil diameter across the trials within each condition at the subject level (C+, C−, NC+, NC−). We had initially preregistered a shorter baseline period of 0.5 s but this shorter time window reduced the numbers of valid gaze samples which resulted in a reduced number of trials to be included in our main analyses (with ensuing reduced statistical power).

We conducted the same analysis for the control trials, where the final frame also remained on the screen for 5 s. In the control condition, the percentage of change in pupil diameter was calculated relative to the pupil diameter during the second movement of the object (used as baseline; 5.1–8.1 s). Given that this baseline is different from the baseline of the experimental trials (because there was no identical still frame and we aimed for a baseline of 3 s to include as many of the control trials as possible), the overall magnitude of changes in pupil dilation is not directly comparable between the experimental and control trials. We therefore analyzed control blocks and experimental blocks separately. Importantly, this choice in baseline could explain overall differences in pupil diameter change across control and experimental trials but it does not explain condition differences within the control and experimental phase.

#### Looking time analyses

For the looking time measure, we added the fixation times of all fixations that fell onto the final scene (AOI surrounding all characters and the object) within each valid trial and averaged the fixation times across all valid trials, separated by condition (C+, C−, NC+, NC−). This looking time approach and measure (i.e., for a 5 s still frame) in a repeated trial design, was adopted from Köster and colleagues^15^ (see also^17^), where the authors successfully observed the hypothesized condition differences in looking times. In line with these prior studies, there was no minimal looking time criterion, and all trials with at least one fixation during the final scene were included. We conducted the same analysis for the control trials.

#### Additional analyses

To further validate that our main finding, that the Action × Response interaction in the experimental trials was robust and reproducible (i.e., not relying on the pupillary surprise response of a few subjects in the whole sample), we assessed a split-half reliability by splitting the sample. We sorted all subjects that remained in the final analysis according to an “ABBA-scheme” and calculated two independent ANOVAs for these samples. Although this is not the standard application of a split-half reliability (i.e., testing the reliability between two test halves at the participant level), we checked this reliability because we deemed it crucial to provide additional information on the robustness of the results.

Finally, we explored the time course of the pupillary response for expected versus unexpected events. To this end, we resampled the recorded fixations to 0.01 s bins and interpolated all missing data (NaNs) using the *inpaint_nans.m* function (mathworks.com/matlabcentral/fileexchange/4551-inpaint_nans), with the default interpolation method (discrete Laplacian; resulting in smoother transitions between data points than a linear interpolation). We interpolated the data because infants did not continuously look at the screen for the entire 5 s that the final scene was presented (see Table S1). The interpolation was applied to the data of each trial before each trial was baseline corrected. The baseline corrected time series of each trial were first averaged across all trials of one condition and then across the expected and the unexpected conditions, respectively. Note that this data does not provide any additional empirical information and was not statistically analyzed. It merely serves to illustrate the temporal change of the pupillary surprise response. We also provide the time course for all four conditions in the supplemental materials (Fig. S3).

We further tested if the action of the third character was associated with the action of the other two characters. For this purpose, we compared the number of referential gazes during the action of the third character, namely if infants looked at the two other characters when the third character performed the same (C) in contrast to a different (NC) action. Specifically, we took the percentage of trials with referential looks, showing at least one fixation on the third character and one fixation to the other two characters during the action or shortly thereafter (24.4–28.4 s). This included again all valid trials with a conform or a non-conform action, respectively. Although this analysis was not preregistered, we included it as a basic check if infants noticed the relation between the action of the third character and the other two characters.

#### Inclusion criteria

Infants were included in the final analysis if they provided one valid trial in each of the four experimental conditions for the pupil dilation or looking time analyses. Trials were included if we recorded one fixation to the screen during the action performed by the first or the second character and during the action performed by the third character, into the region of interest where the actions took place. In addition, for the pupil dilation measure, at least one fixation had to be recorded during the baseline and final scene, whereas for the looking time measure, at least one fixation had to be recorded at the final scene. Note that we set as a minimal criterion that infants needed to watch the first or the second action of the build-up, because it was sufficient for the third action to be ‘conform’ or ‘non-conform’ with the prior behavior and to lose as few trials as possible. Overall, *n* = 29 subjects were included in the pupil dilation analyses and *n* = 30 subject remained for the looking time analyses, providing *M* = 9.8 (*SD* = 3.9) and *M* = 10.4 (*SD* = 3.7) experimental trials, respectively. The initial trial numbers with at least one fixation on the screen were *M* = 14.5 (*SD* = 4.7) and *M* = 14.3 (*SD* = 4.8), for the samples included in the pupillary and looking time analysis, respectively.

In the control analyses, mirroring the experimental trials, it was required that we recorded one fixation to the screen during the first or the second movement and during the third movement, into the region of interest where the movements happened. In the final sample, *n* = 19 infants remained with one trial in each condition for the pupil dilation analyses and *n* = 20 for the looking time analyses.

### Supplementary Information

Supplementary Information.

Supplementary Movie S1.

Supplementary Movie S2.

Supplementary Movie S3.

Supplementary Movie S4.

## Data Availability

The data underlying the main analyses, as well as exemplary stimulus materials are available via the Open Science Framework: https://osf.io/ykz2m/.
