# Supplementary material for: Preverbal infants’ understanding of social norms
Source: Sci Rep. 2024 Feb 5;14:2983. doi: 10.1038/s41598-024-53110-3 (PMC10844370; doi:10.1038/s41598-024-53110-3)
Supplement: Supplementary file 1 — Supplementary Information. [file 41598_2024_53110_MOESM1_ESM.docx]

**Supplementary Information**

*Table S1.* Changes in pupil diameter (in percent from baseline) and looking times (in seconds).

|  | C+ | C- | NC+ | NC- |
| --- | --- | --- | --- | --- |
| Experimental |  |  |  |  |
| Pupil Diameter | -1.4 (1.3) | 2.7 (1.3) | 2.3 (1.6) | -0.4 (1.8) |
| Looking Time | 1.68 (.20) | 1.34 (.14) | 1.47 (.14) | 1.39 (.14) |
| Non-social |  |  |  |  |
| Pupil Diameter | 3.6 (1.2) | 3.6 (3.0) | 3.5 (1.3) | 0.1 (2.4) |
| Looking Time | 2.36 (.26) | 2.28 (.30) | 2.10 (.29) | 2.24 (.27) |

*Note: Mean scores are presented together with standard errors in parentheses. C = conform/consistent, NC = non-conform/non-consistent, + = positive reaction, - = negative reaction*


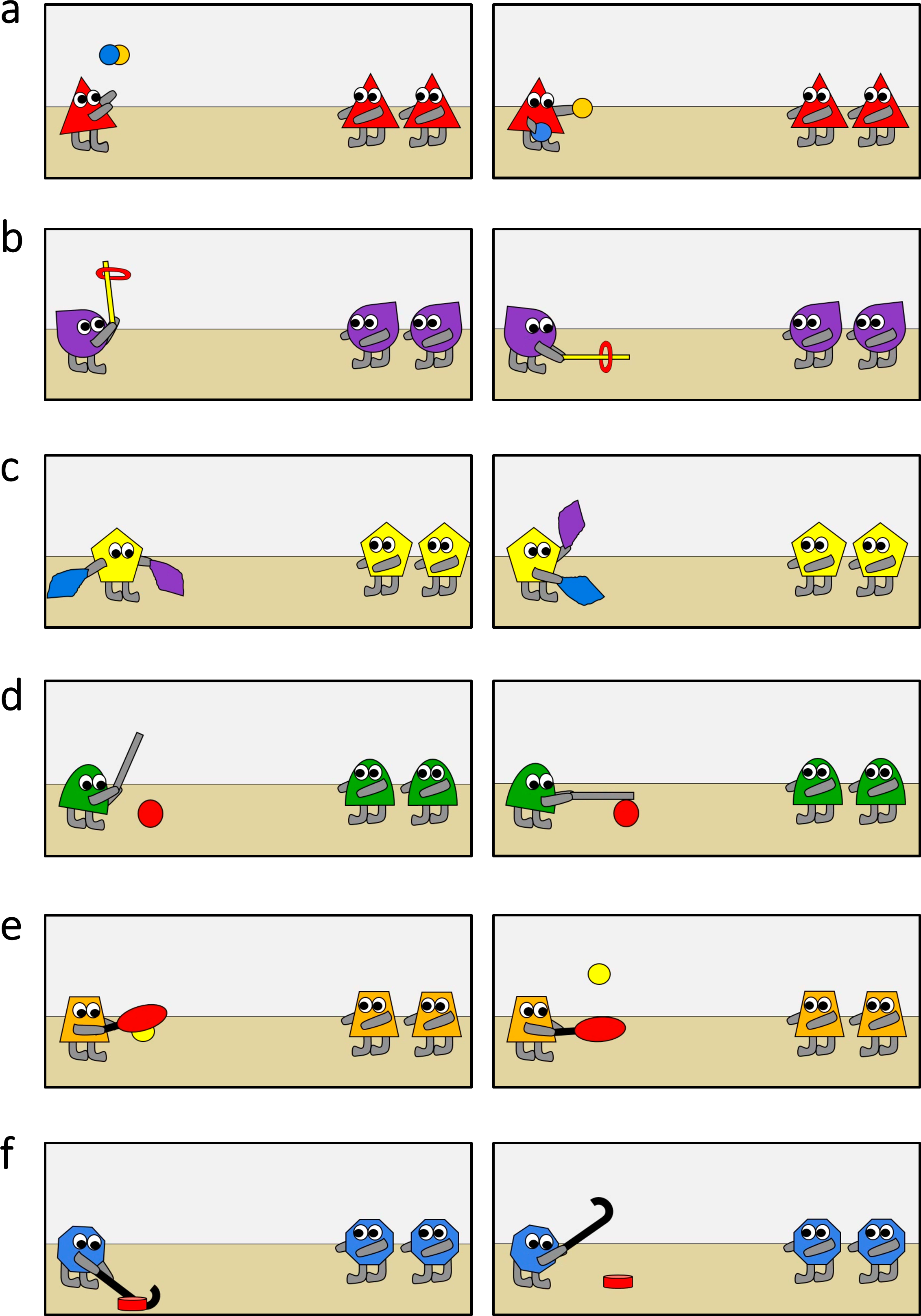


**Figure S1.** Full set of characters and actions shown to the infants. The two different actions which were performed with each object are shown in the right and the left panel. (A) Red triangle characters threw balls up or knocked them together. (B) Purple drop characters lifted a red ring up with a stick or put the stick through the ring. (C) Yellow pentagon characters waved cloths horizontally or vertically. (D) Green knob characters hit or rolled a ball with a stick. (E) Orange trapeze characters smashed a ball up or down with a racket. (F) Blue hexagon characters swiped a disc on the floor or hit it with a cane. All actions were accompanied by sounds. Actions that were shown as the initial action (left or right panel) were counterbalanced between participants. Thereby, we also counterbalanced the action was conform or non-conform.


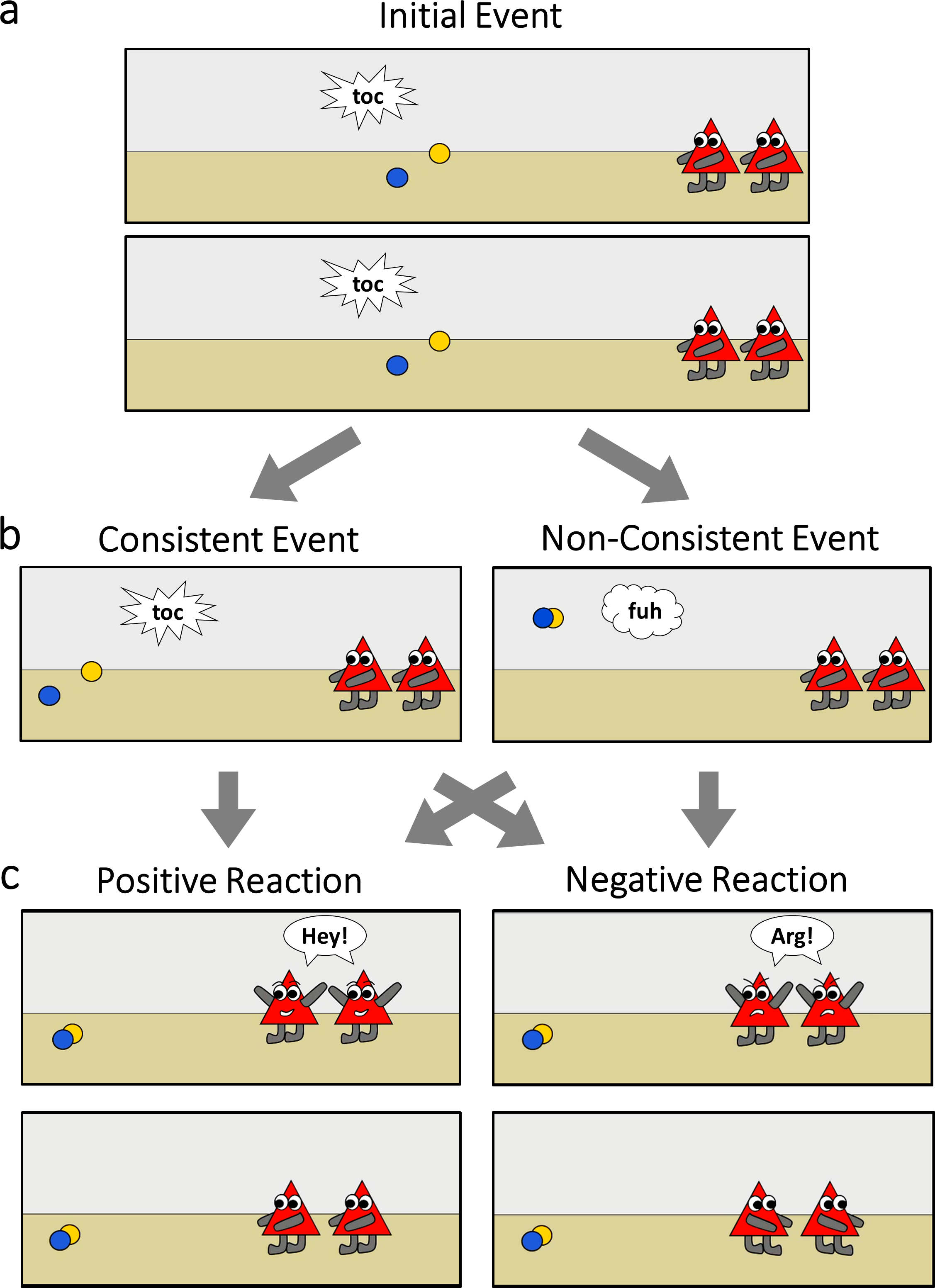


**Figure S2.** Non-social control sequences. (A) In the familiarization phase, two objects made the same movements and sound like the objects in the experimental sequences (cf. Fig. 1), for 2 times, but moved auto-propelled (e.g., 2 balls knocking together, producing a “toc” sound). (B) The third movement of the object was consistent or inconsistent (e.g., the balls moving up in the air, producing a “fuh” sound). (C) The characters moved forward and responded positively (happy expression, “hey” sound, moving towards the individual) or negatively (angry expression, “arg” sound, turning away from the individual). The final scene remained for 5s.


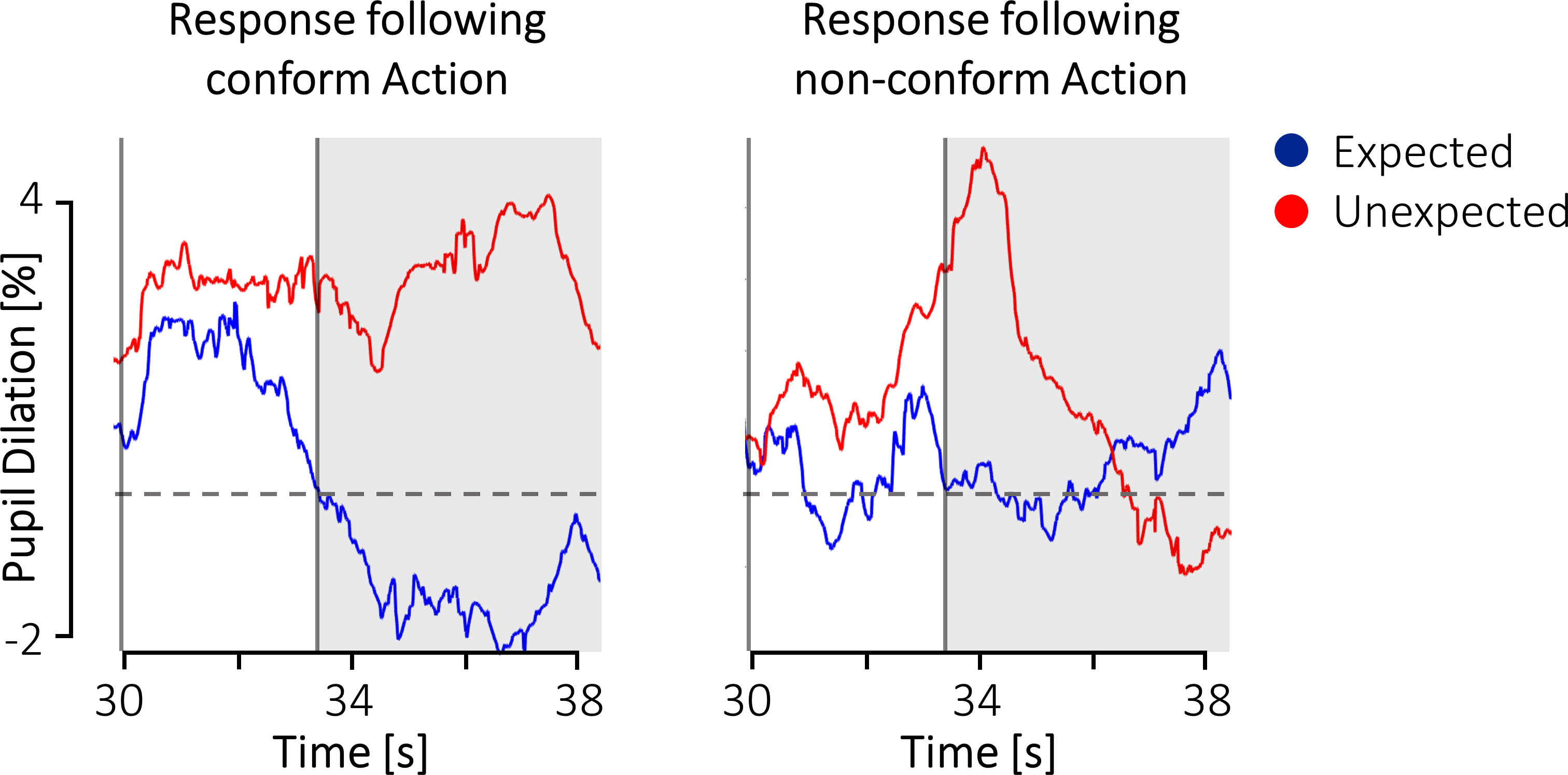


**Figure S3.** The temporal course of the pupillary response in the experimental condition, split by responses following conform and non-conform actions. The time window of the social response is indicated by the white area, corresponding to the period of the expected and unexpected response of the two individuals. The gray area indicates the 5s time window of the final scene, included in the analyses.


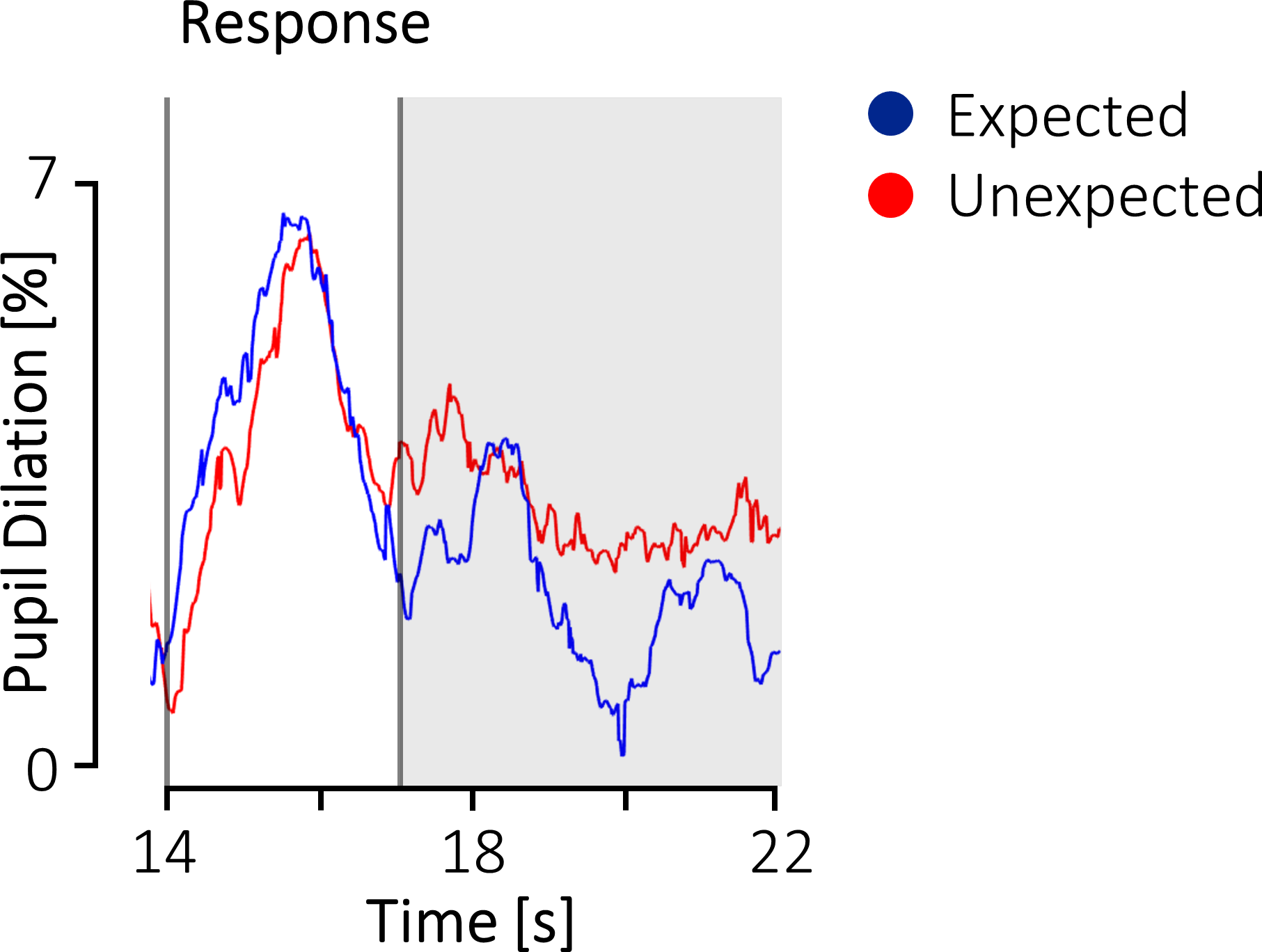


**Figure S4.** The temporal course of the pupillary response in the control condition. The time window of the social response is indicated by the white area, corresponding to the period of the expected and unexpected response of the two individuals. The gray area indicates the 5s time window of the final scene, included in the analyses.
